# Global Effect of Inauhzin on Human p53-Responsive Transcriptome

**DOI:** 10.1371/journal.pone.0052172

**Published:** 2012-12-21

**Authors:** Jun-Ming Liao, Shelya X. Zeng, Xiang Zhou, Hua Lu

**Affiliations:** Department of Biochemistry & Molecular Biology and Tulane Cancer Center, Tulane University School of Medicine, New Orleans, Louisiana, United States of America; Morehouse School of Medicine, United States of America

## Abstract

**Background:**

Previously, we reported that Inauhzin (INZ) induces p53 activity and suppresses tumor growth by inhibiting Sirt1. However, it remains unknown whether INZ may globally affect p53-dependent gene expression or not. Herein, we have conducted microarray and real-time PCR analyses of gene expression to determine the global effect of INZ on human p53-responsive transcriptome.

**Methodology/Principal Findings:**

In this study, we conducted microarray analysis followed by PCR validation of general gene expression in HCT116^p53+/+^ and HCT116^p53−/−^ cells treated with or without INZ. Microarray data showed that 324 genes were up-regulated by ≥2.3-fold and 266 genes were down-regulated by ≥2-fold in response to INZ treatment in a p53-dependent manner. GO analysis for these genes further revealed that INZ affects several biological processes, including apoptosis (GO:0006915), cell cycle (GO:0007049), immune system process (GO:0002376), and cell adhesion (GO:0007155), which are in line with p53 functions in cells. Also, pathway and STRING analyses of these genes indicated that the p53-signaling pathway is the most significant pathway responsive to INZ treatment as predicted, since a number of these p53 target genes have been previously reported and some of them were validated by RT-qPCR. Finally, among the 9 tested and highly expressed genes, ACBD4, APOBEC3C, and FLJ14327 could be novel p53 target genes, for they were up-regulated by INZ in HCT116^p53+/+^ cells, but not in HCT116^p53−/−^ cells.

**Conclusions/Significance:**

From our whole genome microarray analysis followed by validation with RT-qPCR, we found that INZ can indeed induce the expression of p53 target genes at a larger scale or globally. Our findings not only verify that INZ indeed activates the p53 signaling pathway, but also provide useful information for identifying novel INZ and/or p53 targets. The global effect of INZ on human p53-responsive transcriptome could also be instrumental to the future design of INZ clinical trials.

## Introduction

The p53 tumor suppressor inhibits tumor growth not only by transcriptionally regulating the expression of numerous target genes involved in the cell cycle checkpoint control, senescence, autophagy, DNA repair, metabolism, and cell death, but also through transcription-independent pathways [1], [2]. Therefore, during tumorigenesis, most cancers have to shut off the p53 signaling pathway via either mutation of *TP53* or inhibition of wide-type p53 activity [1], [3], [4]. Because of the importance of p53 in anti-tumorigenesis, restoring p53 activity has been one attractive strategy for the development of anti-cancer therapies [5]. Indeed, p53 re-activation or restoration has been shown to regress tumors in different mice models [6], [7], [8]. Although there are still some issues that remain to be solved [6], this approach has been approved to be useful for tumor suppression. Also, since the unique micro-environment in transformed or cancerous cells sometime appears to be required for the activity of p53, restoration of p53 activity in normal cells that lack such an environment often turns out to be insufficient [7], [9], [10]. Thereby, compared to chemotherapy and radiotherapy, p53 restoration might be more effective in specifically targeted tumor cells, but not normal cells, and as such, could provide therapeutic selectivity with minimal side effects on normal cells or tissues.

Over the past decade, several small molecules that can induce p53 level and activity by directly or indirectly targeting this pathway have been identified. Some of them have been put up for early phases of clinical trials, and others are still in the pipeline [11], [12], [13], [14], [15], [16], [17]. These small molecules can be classified into two categories: 1) one that can convert mutant p53 into a functional wild type form; 2) the other that can re-activate wide-type p53 in cancer cells. Among the first category, a small molecule called PRIMA-1 had been shown to render a mutant p53 protein into a form that functions like its wild type version [11]. Also, by screening anticancer drugs, a recent study identified another small molecule named NSC319726 that could specifically convert the R175 mutant p53 into a functional wide-type structure. More remarkably, this compound could inhibit xenograft tumor growth in a mutant p53-dependent fashion [12]. More small molecules have been identified in the second category, including Nutlin, Rita, MI-219, and Tenovins, to activate wild type p53 in cancer cells and to kill them by either directly inhibiting the interaction between MDM2 and p53 or indirectly inducing p53 acetylation [13], [14], [15], [16]. Recently, our group also discovered a new small molecule named Inauhzin (INZ), which induces the level and activity of wide-type p53 by inhibiting Sirt1 activity and also represses the growth of tumors derived from human lung non-small cell carcinoma H460 and colon cancer HCT116 cells in a p53-dependent fashion [17]. Interestingly, we also found that INZ could synergize the anti-cancer effect of Nutlin-3 by cooperating with this inhibitor of the MDM2-p53 interaction in activation of p53 [18].

Although we have learnt that INZ is a p53-dependent anti-cancer agent [17], it remains unclear if this small molecule could have a relatively global effect on the expression of a large group of p53 target genes, including those known and possible unknown target genes. INZ can induce p53 acetylation that is believed to be indispensable for p53 activation and to control the selectivity of p53 targets [19], [20], [21], [22], [23], and there has not been any p53 signature profiling that is induced in response to acetylated p53. Therefore, we were also curious about whether novel p53 targets could be identified in response to INZ-induced p53 acetylation. To this end, we performed a set of microarray and bioinformatic analyses of gene expression in HCT116^p53+/+^ and HCT116^p53−/−^ cells that were treated with or without INZ. Some representative known and novel p53 target genes were also validated by using real-time PCR. Our results not only verify the p53-dependent INZ signature of human transcriptome, but also reveal some potential new p53 target genes that might be activated in response to acetylated p53. This information could also be useful to the future plan for possible INZ clinical trial as an anti-cancer therapy.

## Materials and Methods

### Cell Lines

HCT116 human colon adenocarcinoma HCT116^p53+/+^ or HCT116^p53−/−^ cells were obtained from the Johns Hopkins University Cell Center. HCT116^p53+/+^ or HCT116^p53−/−^ cells were grown in Dulbecco’s modified Eagle’s medium (DMEM) supplemented with 10% fetal bovine serum (FBS), 50 U/ml penicillin and 0.1 mg/ml streptomycin at 37°C in a 5% CO_2_.

### Compounds, Plasmids, and Transient Transfection

INZ was purchased from and verified by ChemBridge Inc., as described previously [17]. The minimum purity of INZ is higher than 90%. Construction of GPF and GFP-p53 was described in [24]. Transient transfection was carried out as described previously [25]. Briefly, cells were transfected with GFP or GFP-p53 plasmids as shown in each figure by using TransFectin (Bio-Rad), following the company’s instruction. Twenty-four hours post transfection, cells were harvested by using TriZol reagents (Invitrogen, Carlsbad, CA).

### RNA Preparation

HCT116^p53+/+^ or HCT116^p53−/−^ cells were treated with 4 µM INZ and harvested at 18 hours post treatment. Cells were then homogenized in TriZol reagents. Three replicates were included for each sample. Total RNA was extracted by following the manufacturer’s standard instructions (Invitrogen). RNA quality was confirmed by agarose electrophoresis and only those samples showing no degradation (ratios approaching 2:1 for the 28 S and 18 S bands) were sent to Arraystar Inc, Rockville, MD for microarray analysis.

### GO Category, Pathway Analysis, and STRING Analysis

Expression data were log2-transformed and normalized against the data for the DMSO treatment control. Heat map was generated by MeV v4.4 [26] (http://www.tm4.org). A gene was selected for this analysis by the following criteria: 1) it must be upregulated at least 2.3 fold or downregulated at least 2 fold by INZ in HCT116^p53+/+^ cells; 2) it was not upregulated or downregulated (less than 1.6 fold) by INZ in HCT116^p53−/−^ cells. GO annotations for the chosen genes were obtained from Gene Ontology (http://www.geneontology.org/). PANTHER was used for the GO enrichment test as previously described [27].

Pathway analysis was performed to determine significant pathways for annotations downloaded from KEGG (http://www.genome.jp/kegg/). DAVID Bioinformatics Resources 6.7 was used to search the significant enrichment for any pathways [28] as follows: briefly, the UniGene IDs of all the genes in the same pool were uploaded into this website-based bioinformatics software. The pathways enriched with at least two genes in the same pool were selected for further analysis. The genes enriched in the p53 pathway were highlighted in red through the KEGG pathway analysis. Pathway categories with a p value ≤0.05 were reported here. Pathway analysis provided a measure for the significance of the function, i.e., with the increased enrichment, the corresponding function is more likely affected by INZ. This analysis helped us identify pathways with greater significance in the experiment.

The chosen genes affected by INZ only in HCT116^p53+/+^ cells were also analyzed by using the protein interactions database, STRING [29] (http://string-db.org/).

### Reverse Transcriptase-polymerase Chain Reaction (PCR) and Quantitative Real-time PCR Analysis

RT-PCR and quantitative real-time PCR (RT-qPCR) for targeting genes were carried out by following the protocol as described previously [30]. Briefly, RT-qPCR was performed using an ABI 7300 real-time PCR system (Applied Biosystems) and the SYBR Green Mix (Applied Biosystems). Relative gene expression was calculated by using the/C/method and following the manufacturer's instruction. All reactions were carried out in triplicate. The primer sequences used are listed in the Table 1.

**Table 1:** Primer sequences.

GenBank ID	Gene	Forward primer	Reverse primer
BC001601	GAPDH	GATTCCACCCATGGCAAATTC	AGCATCGCCCCACTTGATT
NM_078467	p21	TGTATATTCAGCATTGTGGGAGGA	CTGGACTGTTTTCTCTCGGCTC
NM_006763	BTG2	CCAGGAGGCACTCACAGAGCA	ACCCACAGGGTCAGCTCGCT
BC041143	ACBD4	AGCTGTGCTGAATGGTTGAGGAGT	CGAGCCCTGGGGCCCTACTT
NM_014508	APOBEC3C	CACAGATCAGAAACCCGATGAAGGC	CCTTTCTGCATGACAATGGGTCTCA
NM_031455	CCDC3	ACAGTAGGCTCATGTGCTCCTCGG	TGCCGGTTGCGCTTCTCCAG
NM_007074	CORO1A	GCCCTGATCTGTGAGGCCAGC	GGATCTCCCACACCATGACTGTGCA
NM_001003399	DKFZp451A211	ACGGCTGCGAGAAGACGACAG	TGCCACGCTCCTTGCCTGTG
AK024389	FLJ14327	GCGATTGGCCCTTGCCCTGT	TCTGCAAGGTGGTGGGGGCT
NM_014045	LRP10	CAAATCATGCTTGTGAGGACCCCC	CGCTCTGAGCCACAGGCCAG
NM_032853	MUM1	TTGGCCCGAACCGCGACTTC	TGTGAGGCTAACGAGGAAGCAATGG
BC005807	SCD	GCTGTGGGTGAGGGCTTCCAC	CTGGCCAAGATGGCGGCCTT

## Results

### Global Changes of Gene Expression in Cells Treated with INZ

**Figure 1:**
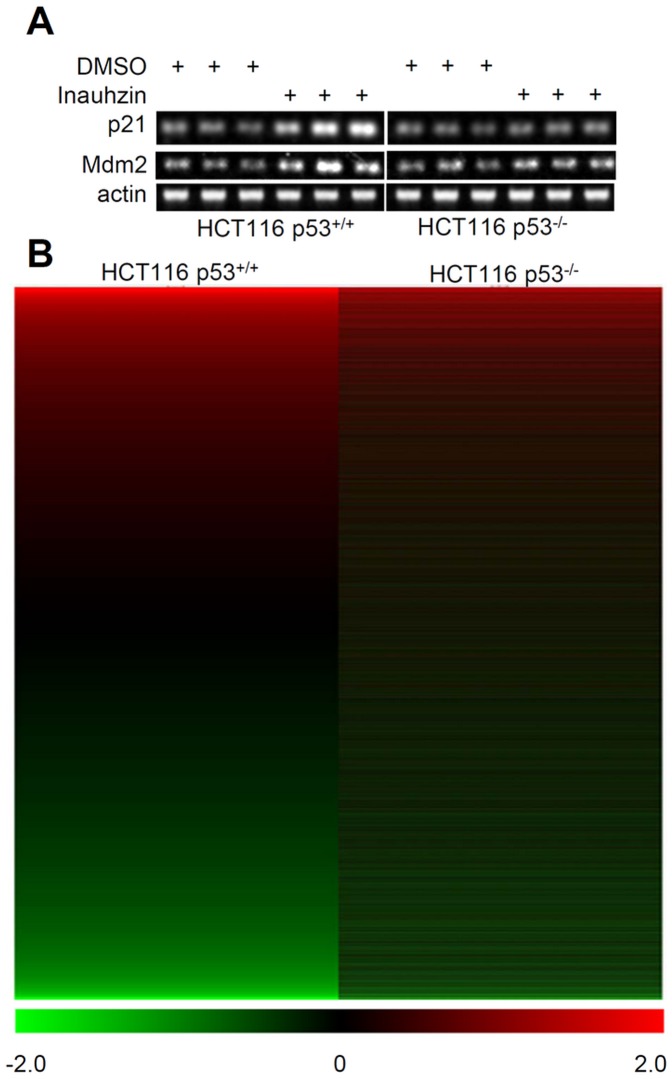
Global transcript profiles in HCT116^p53+/+^ and HCT116^p53−/−^ cells in response to Inauhzin treatment. (**A**) Inauhzin (INZ) increased p21 and mdm2 mRNA in HCT116^p53+/+,^ but not in HCT116 ^p53−/−,^ cells. Cells were treated with 4 µM INZ for 18 hours and harvested for RT-PCR. RT-PCR products of actin were used as an internal control. (**B**) Overview of the entire transcript profiles after INZ treatment in HCT116^p53+/+^ and HCT116^p53−/−^ cells. The heat maps display the gene expression by the log2-transformed fold changes as normalized with DMSO treatment. Green indicates lower expression; red indicates higher expression; black is for the log2 (fold change)  = 0. All samples were triplicated as shown in panel A.

We previously showed that INZ increases p53 level and activity by inhibiting Sirt1 activity [17]. However, it remains unclear whether INZ globally affects the expression of p53 target genes and whether there are p53-independent, but INZ-responsive, target genes. To address these questions, we conducted a set of microarray analyses of gene expression in HCT116^p53+/+^ or HCT116^p53−/−^ cells treated with DMSO or 4 µM INZ. These cells were treated with INZ under the same condition as that as previously shown [17] and then harvested for total RNA extraction and RT-PCR analysis. As expected [17], the p53 activity as measured by the expression levels of p21 and MDM2 mRNAs was confirmed to be responsive to INZ treatment (Figure 1A). The microarray analysis of the same mRNA samples revealed the expression of 25,707 genes, 11,704 of which displayed valid expression values from this analysis. Approximately 10.8% and 11.3% of the genes were up- or down-regulated in HCT116^p53+/+^ and HCT116^p53−/−^ cells, respectively, while the expression of the rest 89% of the genes was not significantly affected by INZ in light of their P values (P≥0.05) and fold changes (<2). Approximately one hundred of genes showed either over 3-fold induction or 70% reduction by INZ in both of the cell lines. A clustering analysis was also performed for the whole set of probes (over 25,707 genes) using the MeV v4.8.1 package. As shown in Figure 1B, INZ significantly induced or reduced the expression of p53-dependent target genes in HCT116^p53+/+^ cells, though it could also alter the expression of some p53-independent target genes in HCT116^p53−/−^ cells to a much lesser degree. This result confirms that INZ indeed is a p53 activator. Therefore, this study only focused on further characterization of p53-dependent target genes in order to gain detailed information about the global effect of INZ on the p53 signaling pathway and also to identify potential novel p53 target genes.

### Identification of p53-dependent Differentially Expressed Genes

**Figure 2:**
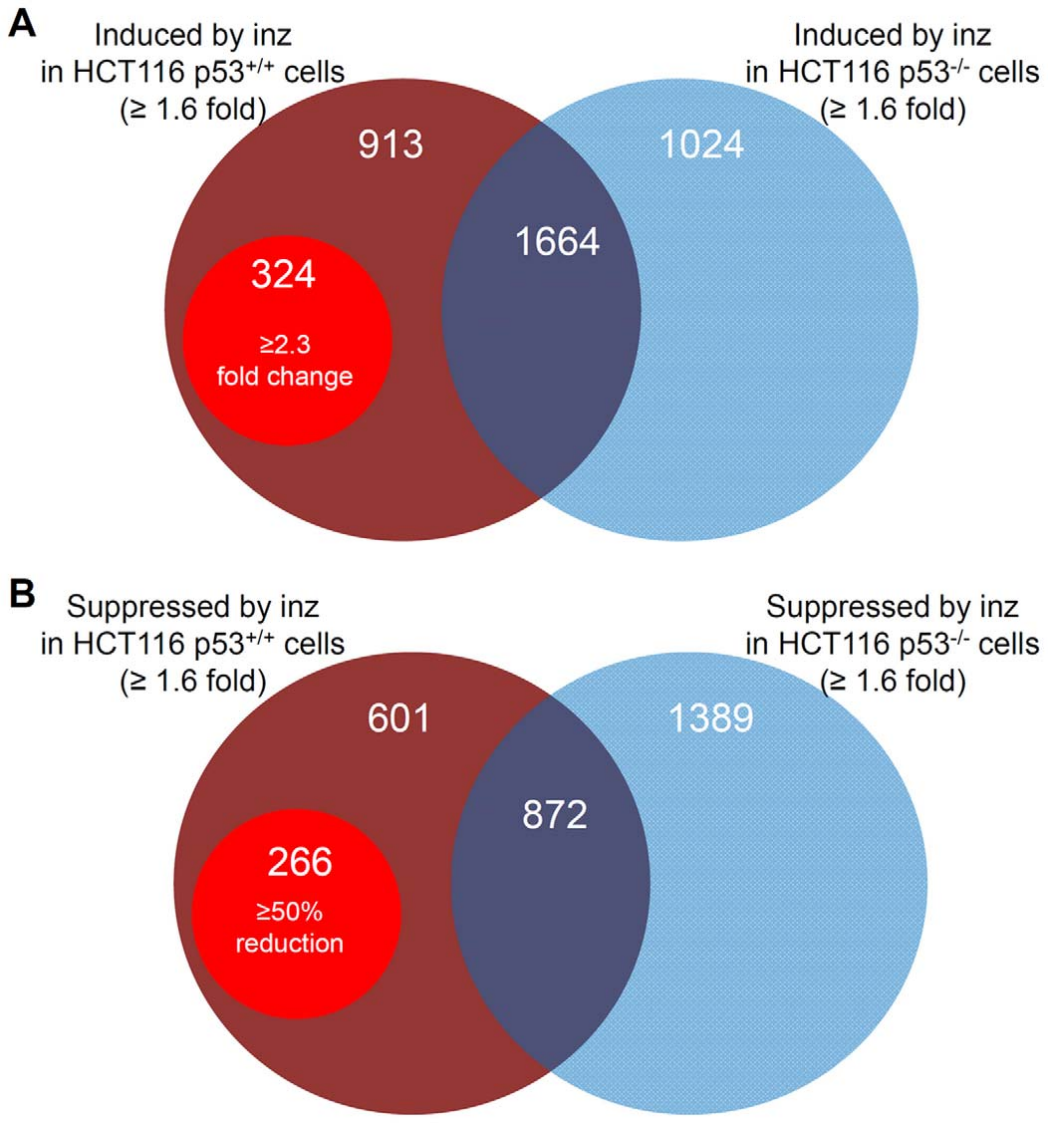
Venn diagram of genes regulated by Inauhzin. Only those genes that observed at least 1.6-fold up (**A**) or down (**B**) regulation in comparison with the control were included in this figure. 1267 and 867 genes were up- and down-regulated, respectively, by INZ in HCT116^p53+/+^ cells. Those genes that meet the indicated cut-off (2.3-fold induction or 50% reduction) are shown in the red circles.

The p53-dependent differentially expressed genes (DEGs) should be those genes that are regulated by INZ in HCT116^p53+/+^ cells, but not in HCT116^p53−/−^ cells. Thereby, from the whole set of the probes on the chip, we first picked up 2901 genes that were up-regulated by INZ (≥1.6 fold) in HCT116^p53+/+^ cells. Among them, 1237 genes were not induced by INZ in HCT116^p53−/−^ cells, suggesting that the rest of the genes (2901–1237 = 1664) are p53-dependent DEGs. To further exclude non-specific target genes, we only selected those genes whose levels were altered more than 2.3-fold compared to the control in this pool. As a result, 324 genes were identified to be significantly induced by INZ as p53-dependent DEGs (Figure 2A). By using the same strategy to screen the INZ-down-regulated genes, we also found 266 p53-dependent DEGs that were suppressed by >50% in response to INZ treatment (Figure 2B). In conclusion, we have identified a total of 590 p53-dependent DEGs that were responsive to INZ treatment in HCT116^p53+/+^ cells, but not in HCT116^p53−/−^ cells.

### Annotation and Functional Analysis of p53-dependent DEGs

**Figure 3:**
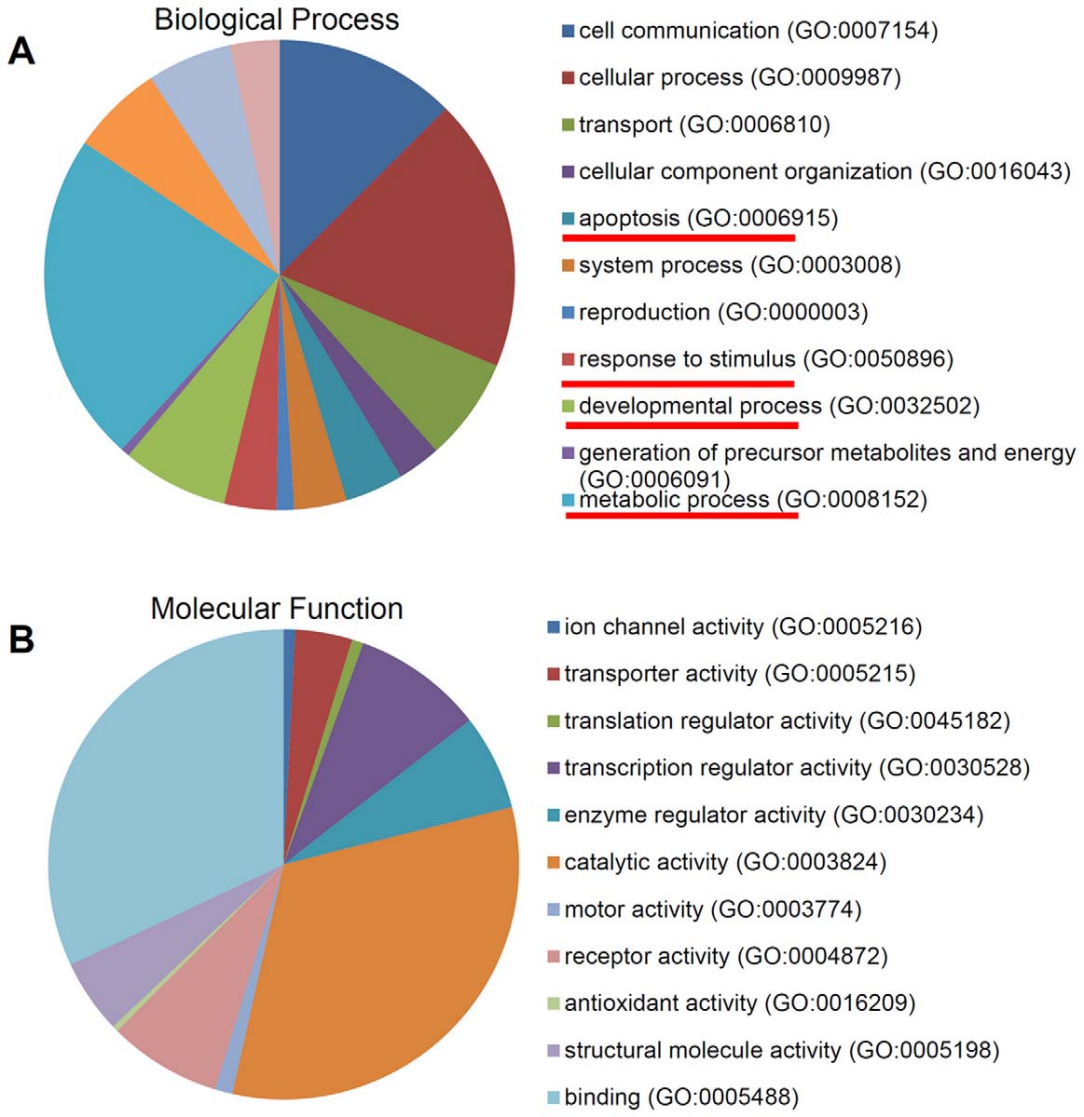
GO analysis of 324 genes up-regulated by Inauhzin only in HCT116^p53+/+^ **cells.** (**A**) and (**B**) Pie charts show the percentage of up-regulated genes, which were categorized based on their involvement in biological processes (**A**) and molecular functions (**B**).

**Figure 4:**
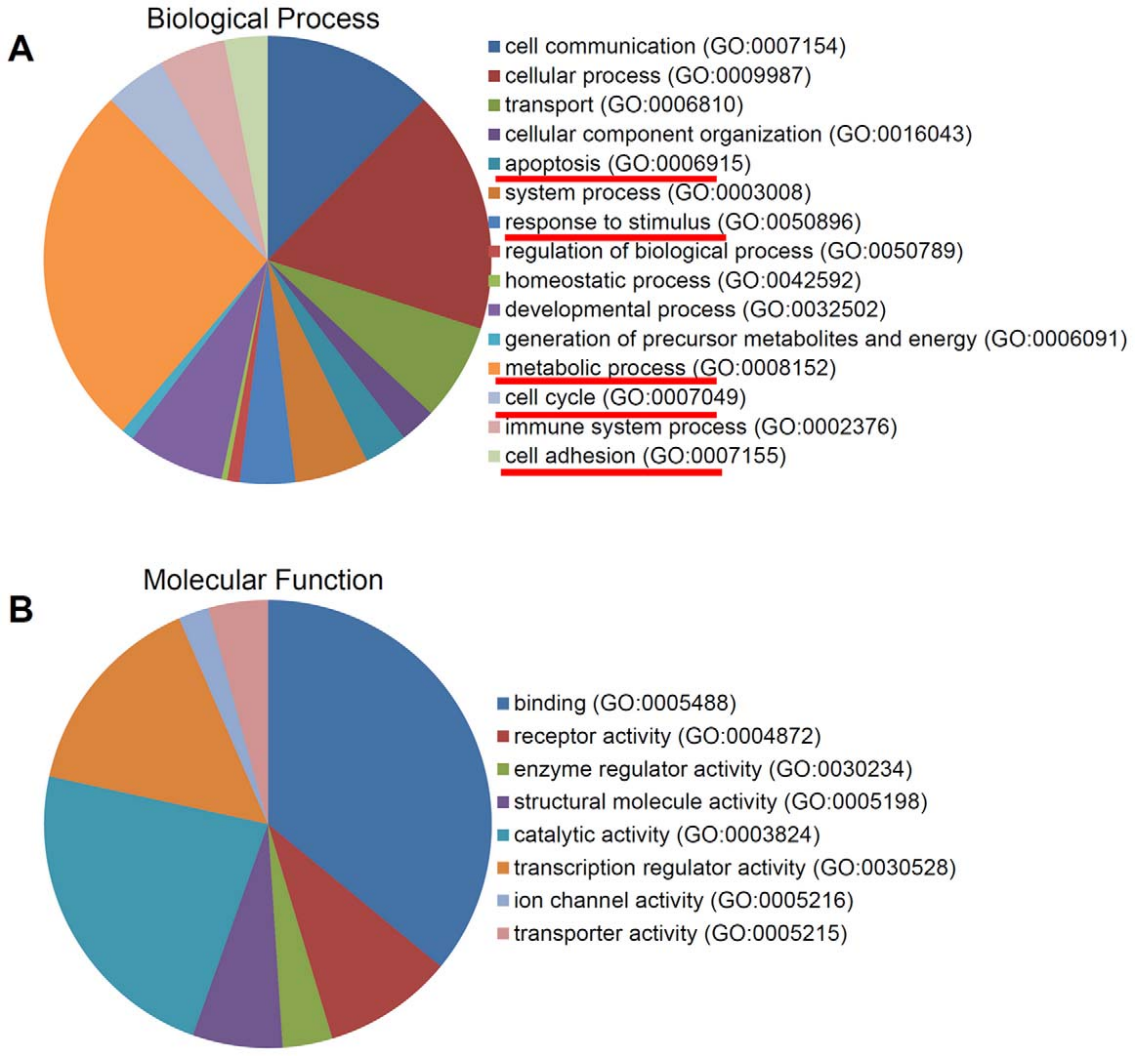
GO analysis of 266 genes down-regulated by Inauhzin only in HCT116^p53+/+^ **cells.** (**A**) and (**B**) Pie charts show the percentage of downregulated genes, which were categorized based on their involvement in biological processes (**A**) and molecular functions (**B**).

The up-regulated and down-regulated DEGs were independently subjected to analysis using a website based database, PANTHER [27] to determine the role of each gene in biological processes and molecular functions. The top GO categories for up-regulated genes include apoptosis (GO:0006915), response to stimulus (GO:0050896), metabolic process (GO:0008152), cell cycle (GO:0007049), immune system process (GO:0002376), and cell adhesion (GO:0007155) (Figure 3A and B), most of which have been reported to be associated with p53 functions in cells. Interestingly, these GO categories were also among the top GO categories for down-regulated genes (Figures 4A and B). These results indicate that the p53 pathway indeed plays a major role in cellular response to INZ treatment.

### KEGG Pathway Analysis of p53-dependent DEGs

**Figure 5:**
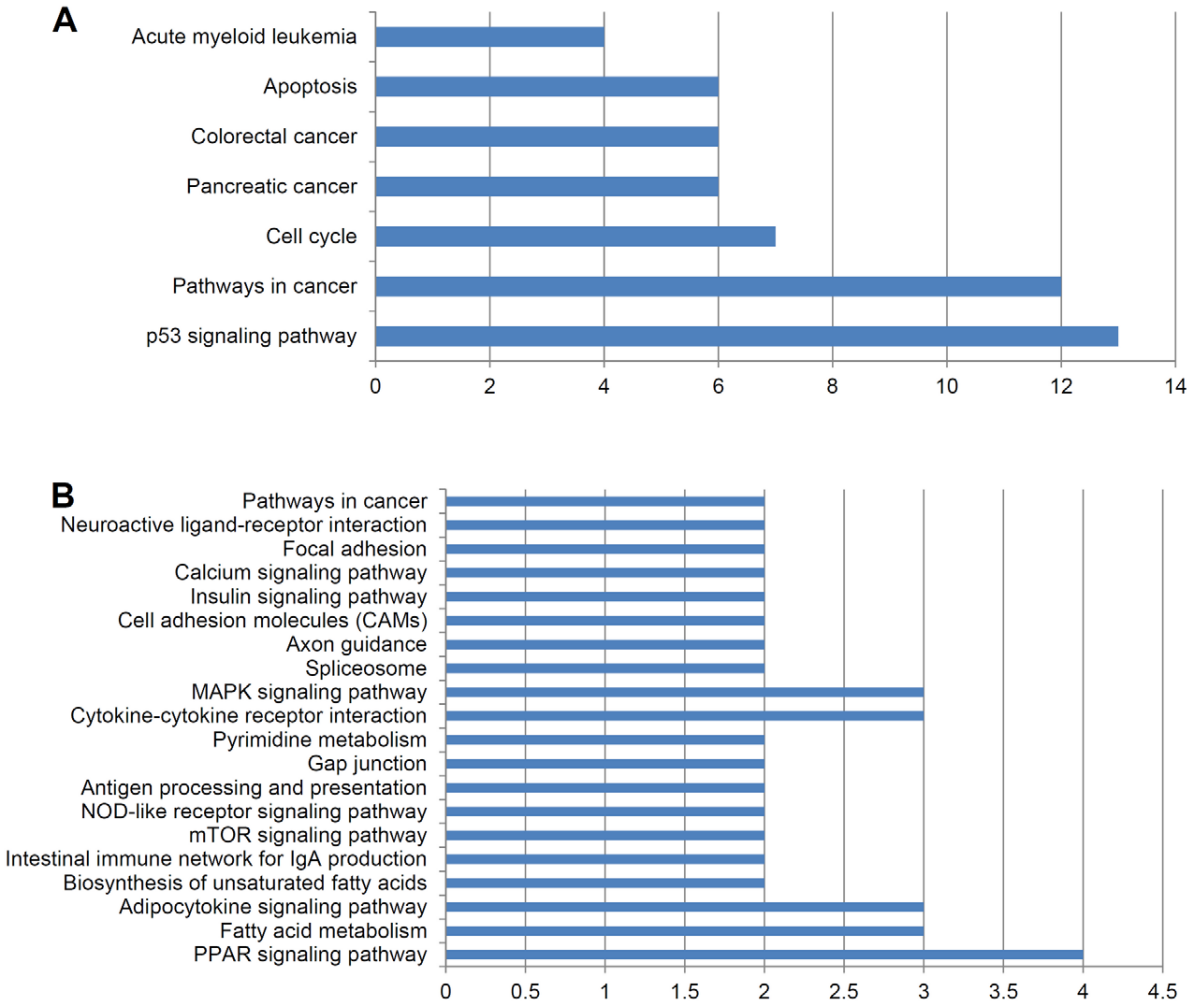
KEGG pathway analysis of 324 genes up-regulated and of 266 genes down-regulated by Inauhzin only in HCT116^p53+/+^ cells. (**A**) The significant pathways for INZ-up-regulated genes. (**B**) The significant pathways for INZ-down-regulated genes.

**Figure 6:**
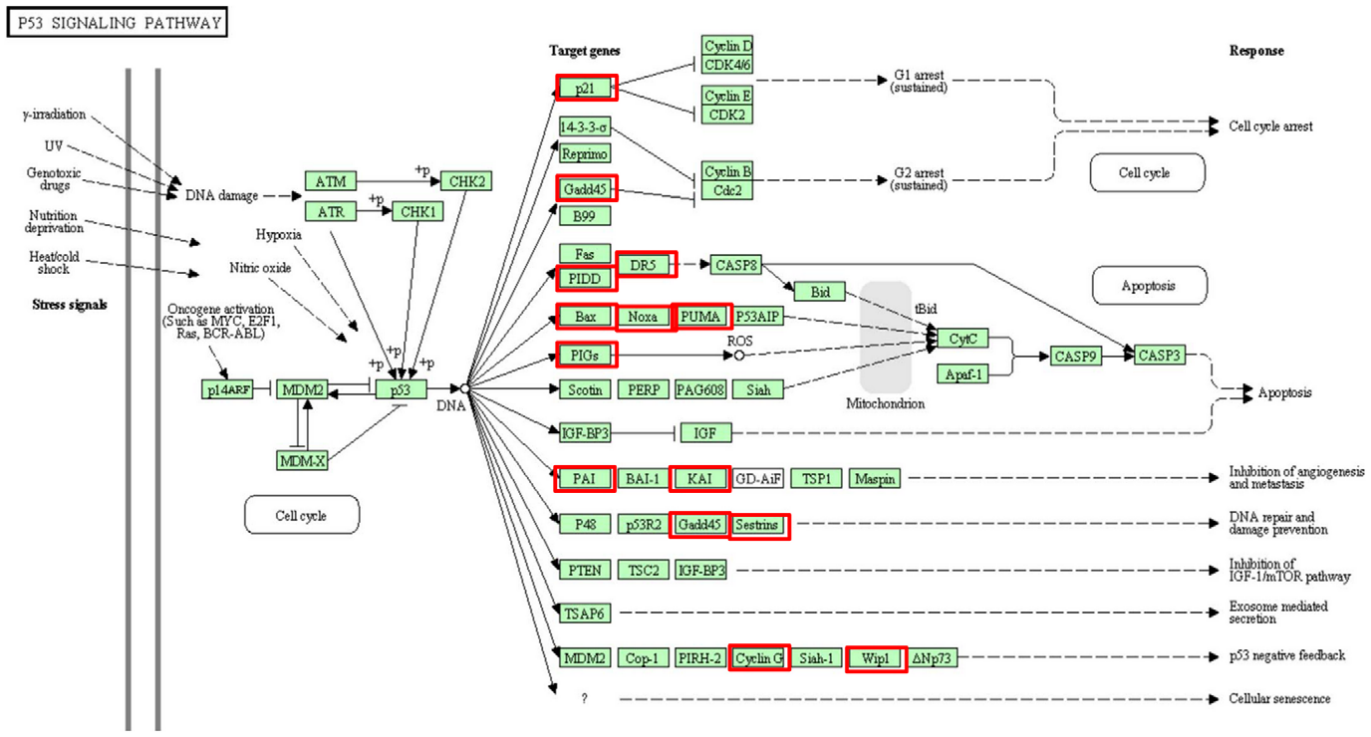
Genes induced by INZ in p53 pathway. The p53 signaling pathway identified by Pathway-Express analysis. Pathway-Express analysis was performed on the INZ-up-regulated genes. Among the most significant KEGG pathways predicted to be relevant to NZ treatment was the p53 signaling pathway. In red square are the genes that were up-regulated by INZ.

DAVID Bioinformatics Resources 6.7 was used to search for pathways enriched with p53-dependent DEGs [21]. The KEGG pathway analysis for up-regulated DEGs showed that 13 DEGs are enriched in the p53 signaling pathway, which is actually the top pathway for up-regulated DEGs. Intriguingly, other pathways for up-regulated DEGs included pathways in cancer, cell cycle, pancreatic cancer, colorectal cancer, apoptosis, and acute myeloid leukemia, which are all related to cancer (Figure 5A). However, the down-regulated DEGs were involved in pathways that include the PPAR signaling pathway, Fatty acid metabolism, and the MAPK signaling pathway (Figure 5A and B). In addition, thirteen p53 direct target genes, involved in cell cycle arrest, apoptosis, inhibition of angiogenesis and metastasis, DNA repair and damage prevention, and p53 negative feedback, were regulated by INZ as summarized in Figure 6. This pathway analysis further demonstrates that INZ is a bona-fide p53 activator and affects the expression of most of the p53 downstream molecules.

### STRING Analysis of the Connections between p53-dependent DEGs

**Figure 7:**
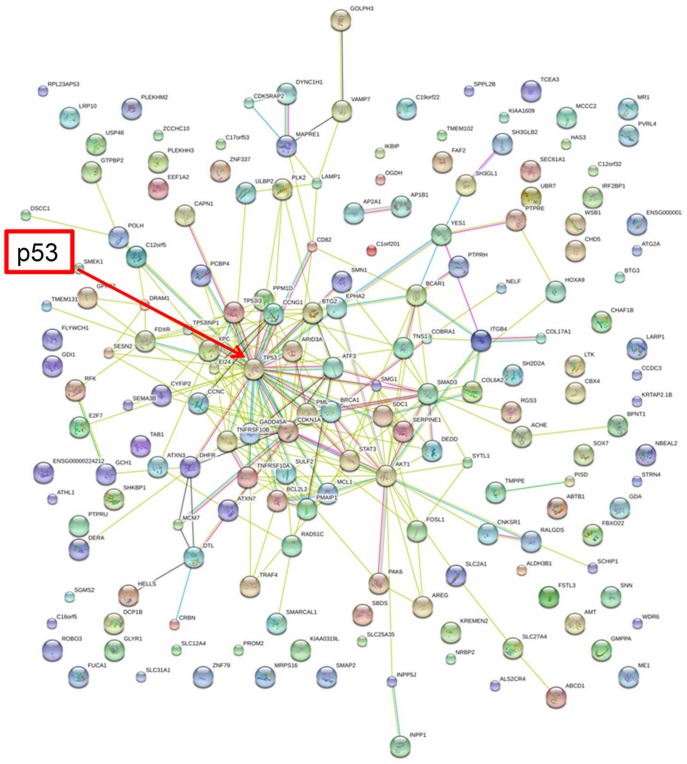
STRING analysis shows that p53 is involved in cell response to Inauhzin treatment. STRING analysis of 324 genes up-regulated by INZ only in HCT116^p53+/+^ cells. The network nodes stand for that genes affected by INZ as shown in red cycle in Figure 2. Lines in different color represent 7 types of evidence used in predicting associations. Red line: fusion evidence; green line: neighborhood evidence; blue line: coocurrence evidence; purple line: experimental evidence; yellow line: text mining evidence; light blue line: database evidence and black line: co-expression evidence.

**Figure 8:**
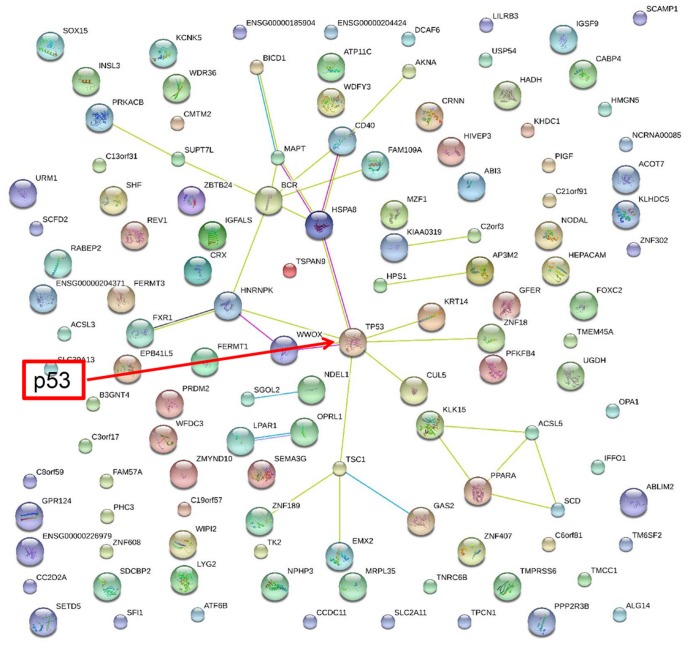
STRING analysis of 266 genes down-regulated by INZ only in HCT116^p53+/+^ cells. The network nodes stand for that genes affected by INZ as shown in red cycle in Figure 2. Lines in different color represent 7 types of evidence used in predicting associations. Red line: fusion evidence; green line: neighborhood evidence; blue line: coocurrence evidence; purple line: experimental evidence; yellow line: text mining evidence; light blue line: database evidence and black line: co-expression evidence.

Up-regulated and Down-regulated DEGs were uploaded separately with p53 into STRING (http://string90.embl.de), a database for known and predicted protein interactions, to determine the direct and indirect connections between these genes and p53. As shown in Figure 7, p53 was the major node in this network, and most of the up-regulated DEGs were associated with p53 either through direct or indirect connections. For example, INZ-up-regulated gene, INPP1, could connect to p53 through INPP5J, AKT1, STAT3, and CDKN1A (Figure 7). However, STRING analysis of Down-regulated DEGs showed that only a few genes in this pool are linked with p53, indicating that their functions might not be related to p53 or currently unknown (Figure 8). This could be due to the possibility of that less attention has been paid to p53-downregulated genes than to p53-upregulated genes. Alternatively, many of these down-regulated DEGs could be regulated via p53 responsive miRNAs [24], [31], [32], [33], [34], [35], [36], [37], [38], [39], [40], [41], [42], [43], [44], which might not have been integrated into this software program. In summary, these data suggest that most of the INZ up-regulated DEGs are highly associated with p53, validating the regulation of p53 activity by this small molecule.

### Validation of the Expressions of Selected DEGs by RT-qPCR

**Figure 9:**
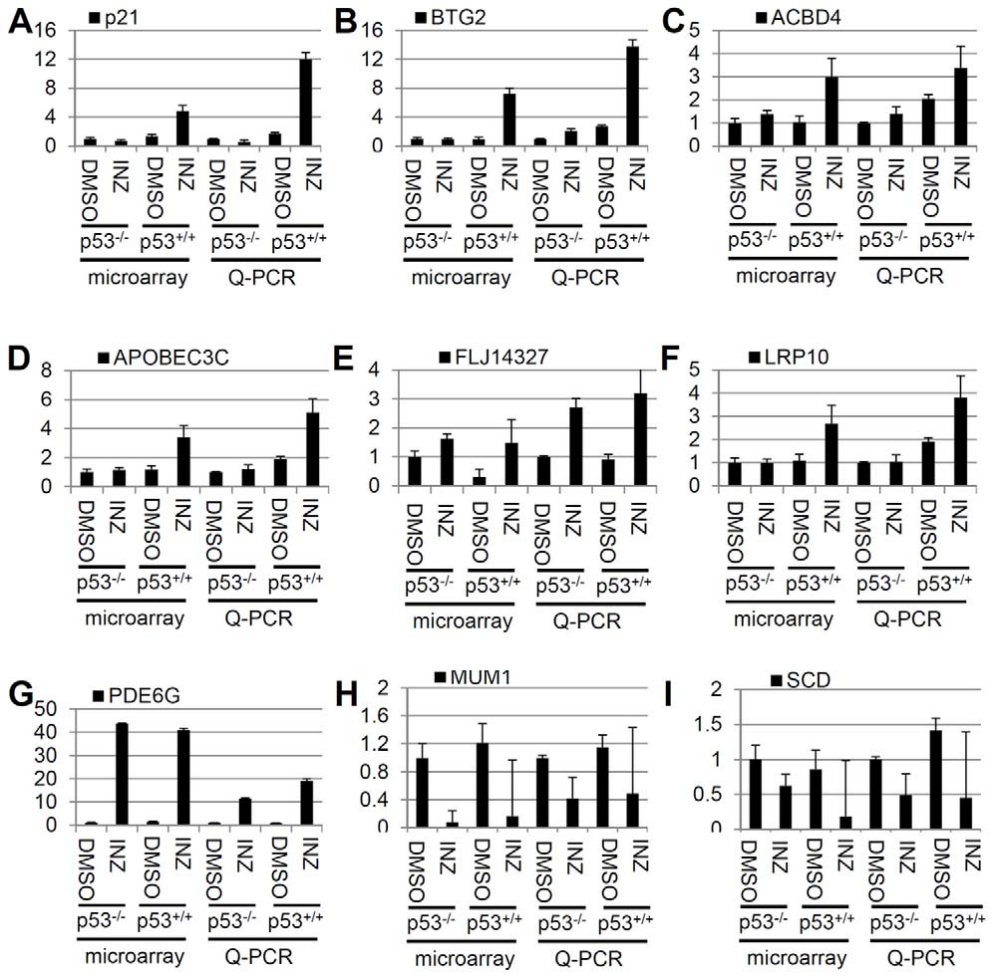
Validation of twelve Inauhzin-regulated genes identified by microarray analysis. Same RNA extracts as those used in Figure 1 were used for RT-qPCR. (**A**) **to** (**F**) Expression of six genes up-regulated by INZ only in HCT116^p53+/+^ cells. (**G**) Expression of PDE6G that was up-regulated by INZ in both HCT116^p53+/+^ and HCT116^p53−/−^ cells. (**H**) **and** (**I**) Expression of MUM1 and SCD that down-regulated by INZ. Data are presented as mean ± standard error, n  = 3.

**Figure 10:**
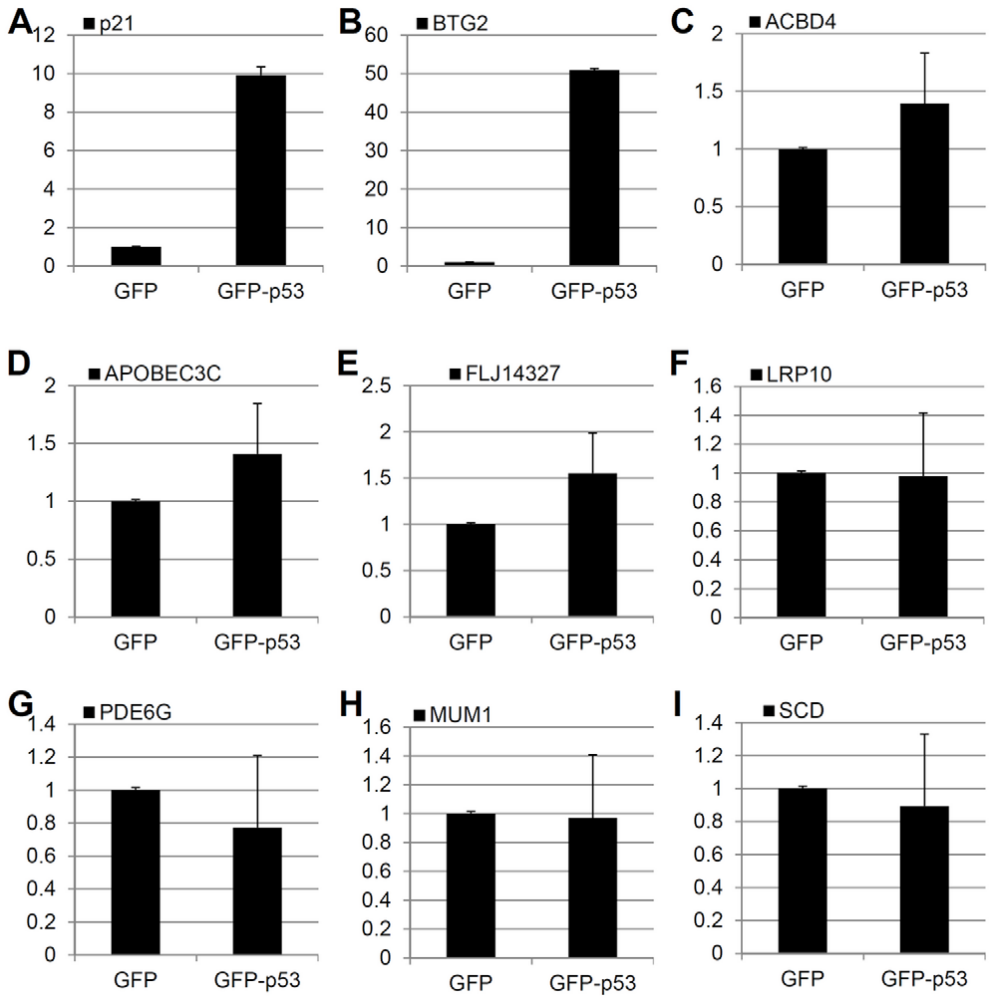
Not every gene induced by Inauhzin is regulated by overexpressing p53. H1299 cells were transfected with GFP or GFP-p53 plasmid and harvested at 24 hours post-transfection. Total RNAs were extracted and subjected to RT-qPCR. (**A**) **to** (**F**) Expression of six genes up-regulated by INZ only in HCT116^p53+/+^ cells. (**G**) Expression of PDE6G that was up-regulated by INZ in both HCT116^p53+/+^ and HCT116^p53−/−^ cells. **H) and** (**I**) Expression of MUM1 and SCD that were down-regulated by INZ. Data are presented as mean ± standard error, n  = 3.

**Figure 11:**
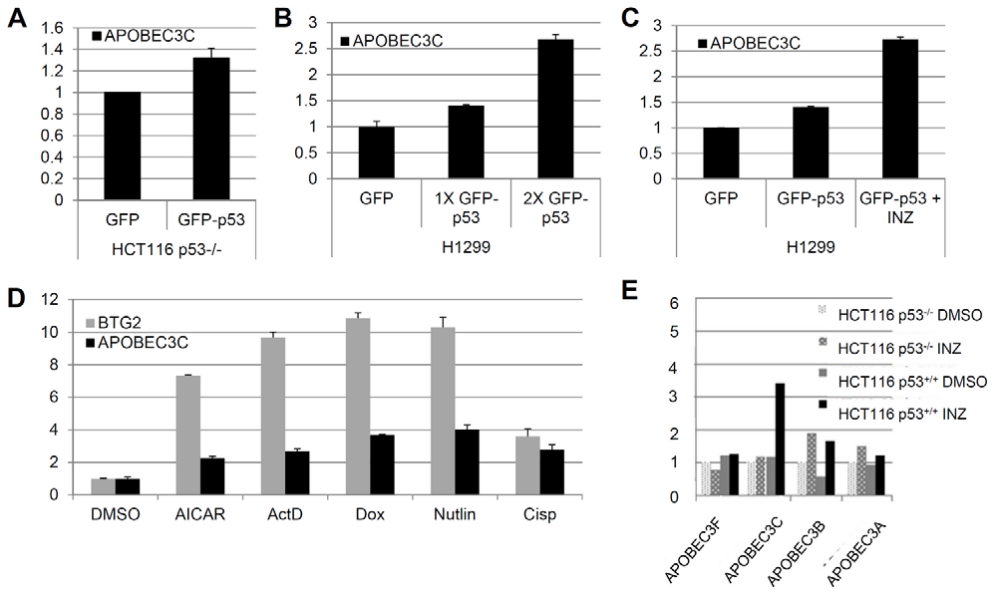
APOBEC3C is induced by p53 activation (A) **Overexpressed p53 induces APOBEC3C mRNA levels in HCT116 p53^−/−^ cells.** HCT116 p53^−/−^ cells were transfected with the GFP or GFP-p53 plasmid and harvested at 24 hours post-transfection. Total RNAs were extracted and subjected to RT-qPCR. (**B**) Overexpressed p53 induces APOBEC3C mRNA levels in H1299 cells in a dose-dependent manner. H1299 cells were transfected with the GFP or GFP-p53 plasmid and harvested at 24 hours post-transfection. Total RNAs were extracted and subjected to RT-qPCR. (**C**) INZ further enhances the induction of APOBEC3C by p53. H1299 cells were transfected with indicated plasmids and treated with or without INZ (2 µg). Total RNAs were extracted and subjected to RT-qPCR. (**D**) APOBEC3C is induced by several p53-activating agents. HCT116 Cells were treated with indicated drugs. Total RNAs were extracted and subjected to RT-qPCR. (**E**) INZ induces APOBEC3C, but not other family members of APOBEC3C, in a p53 dependent fashion. HCT116 and HCT116 p53^−/−^ cells were treated with indicated agents. Total RNAs were extracted and subjected to RT-qPCR.

To further verify the microarray results, we carried out RT-qPCR for 9 selected DEGs, six from the p53-dependent DEG set (Figures 9A–9F) and three from p53-independent DEG set (Figures 9G–9I). The results showed that the induction of these mRNA levels as determined by qPCR is in good accordance with those from microarray analysis (Figure 9). Of note, the expression of the FLJ14327 gene was inconsistent with the microarray data (Figure 9E). This could be caused by either experimental errors or the discrepancy in the sensitivity of detection between these two assays [45], [46], [47], [48]. In order to further validate these data, we also examined the mRNA expression of these DEGs after overexpression of ectopic p53. H1299 cells were transfected with GFP-p53 or GFP plasmid and harvested 24 hours post-transfection. The mRNA levels of these DEGs were determined by RT-qPCR. As shown in Figure 10, in addition to p21 and BTG2 that were previously shown as p53 target genes [49], [50], the expression of ACBD4, APOBEC3C, and FLJ14327 mRNA levels were all induced by ectopically expressed p53, indicating that these genes could be novel p53 targets. However, LRP10, PDE6G, MUM1, and SCD mRNAs were not affected by ectopic p53, suggesting that these genes could be INZ responsive, but p53-independent, targets. As one example for the proof of the principle test here, we checked if ectopic p53, as simply overexpressed in p53 null human cancer cells, such as HCT116^p53−/−^ or H1299 cells, could induce one of these new target genes, APOBEC3C. As shown in Figure 11, we surprisingly found that exogenous p53 could only marginally induce the expression of APOBEC3C mRNA by less than 1 fold (Figures 11A and 11B), but this induction was markedly enhanced by INZ with more than 2 fold (Figure 11C). Consistent with this result, other p53 activating agents as listed in Figure 11D could also induce APOBEC3C mRNA levels in p53-containing HCT116 cells markedly, even though to a less extent compared to that of BTG2, a known p53 target gene [49]. Among the APOBEC3C family members, APOBEC3C was the only one that can be induced by INZ (Figure 11E). This preliminary study suggests that some of the p53 target genes might be responsive to modified and activated p53 rather than just its simply elevated level as further discussed below. Together, these results indicate that overall the DEGs identified through microarray analysis can be validated by RT-qPCR as p53-dependent target genes, although a small portion of them might be microarray artifacts or cell-specific targets for p53.

## Discussion

It has been known that p53 can induce the expression of numerous target genes that are important for various p53-dependent cellular activities or functions, such as cell death, cell cycle arrest, DNA repair, autophagy, senescence and auto-regulation [2]. However, the timing and the extent of their expression vary since not all of these cellular activities would occur simultaneously upon p53 activation in response to each type of stress or distinct stress signals. Also, selective expression of certain p53 target genes in response to certain stress is highly associated with cellular outcomes under this specific stress. Although the detailed mechanisms underlying the selection of certain p53 target genes in response to a certain type of stress signals still remain elusive, p53 acetylation at different lysine sites has been shown to play an important role in this selection in response to DNA damage signals [1]. For example, acetylation of p53 at K320 is required for transcription of some apoptosis-related target genes, but does not affect p21 transcription [51], [52]. Because INZ induces p53 acetylation [17], INZ may activate the expression of p53 target genes that could be more likely involved in apoptosis than in the cell cycle regulation. Indeed, among the 324 p53-depedent DEGs by INZ, approximately 20–25% of them are involved in apoptosis, whereas only about 5% of them are involved in the cell cycle regulation (Figure 3A). This finding is in line with the notion that acetylated p53 might preferentially activate the expression of pro-apoptotic genes [1] and also consistent with our previous report showing that one major cellular phenotype in response to INZ treatment is the p53-dependent apoptosis [17]. In addition, our microarray analysis also revealed some novel p53 target genes, such as ACBD4, APOBEC3C, and FLJ14327 (Figures 9 and 10), which would not be identified by simply overexpressing ectopic p53 in cancer cell lines [20], [53], because INZ-activated p53 is acetylated [17], although further studies of these new p53 target genes are necessary to determine whether and how these genes, such as APOBEC3C (Figure 11), might act in the p53 signaling pathway.

In addition to those potential p53 target genes, we also identified hundreds of genes that were regulated by INZ regardless of p53 status (Figures 1 and 2). These genes could also be of interest for multiple reasons. First, novel INZ target genes that are related to cancer development might be identified from this pool. Second, analysis of these genes could be conducive to our better understanding of the possible adverse effects of INZ. As almost every drug has off-targets [54], [55], an overview of INZ off-targets is vital for our future designing INZ derivatives with minimum side effects. Last, analysis of these genes might help us fully understand how INZ induces p53 levels in cells and why INZ is less toxic to normal cells or tissues [17]. We are also curious about whether INZ could activate p53 via pathways independently of Sirt1. In summary, analysis of these p53-independent DEGs could offer a better picture about how INZ functions in cells.

Interestingly, we also identified 266 genes that were down regulated by INZ in HCT116^p53+/+^ cells, but not in HCT116^p53−/−^ cells (Figure 2). This result suggests that p53 might either inhibit their transcription or regulate the stability of their mRNAs. Indeed, p53 has been shown to suppress gene expression either by direct binding to the promoters [56], [57], [58], [59], [60] or via various miRNAs [44]. Interestingly and surprisingly, STRING analysis of these target genes showed that most of the genes in this pool are not functionally related to p53. This could be due to the following possibilities: 1) less attentions have been paid to p53 down-regulated genes in establishing this bioinformatics program; 2) the p53 target miRNAs, which might mediate the suppression of these target genes, have not yet been identified. Therefore, an interesting project for future study would be to investigate whether miRNAs are involved in the inhibition of these genes by p53 and whether and how these p53-down-regulated genes act in tumorigenesis.

The therapeutic synergy of p53 restoration with other drugs, including DNA-damage agents and other oncoprotein inhibitors, has been previously shown in cell and animal model systems [18], [61], [62], [63]. For example, we recently showed that INZ and Nutlin, another p53 activator by inhibiting the p53-MDM2 interaction [15], can synergistically activate p53 and suppress tumor growth in both cells and animal models [18]. However, although combination of a p53 activating small molecule with a classical chemotherapeutic drug might be more potent than a single treatment, this combined treatment might also cause a synergistic adverse effect on patients. Understanding the global effects of each drug on cells is necessary for designing a better cocktail treatment with minimum side effects. Therefore, our microarray and bioinformatics analyses of whole human cell transcriptome in response to INZ treatment as described in this study not only verifies the notion that INZ indeed is a p53 activator with some novel target genes identified, but also provides useful information for our future design of possible INZ clinic trials.
